# Morphological study of the vastus medialis oblique in recurrent patellar dislocation based on magnetic resonance images

**DOI:** 10.1186/s12880-020-00542-8

**Published:** 2021-01-06

**Authors:** Lei Shu, Xu Yang, Hangyuan He, Biao Chen, Liaobin Chen, Qubo Ni

**Affiliations:** Department of Orthopedic Surgery, Zhongnan Hospital of Wuhan University, Wuhan, 430071 China

**Keywords:** Recurrent lateral patellar dislocation, Vastus medialis obliquus, Magnetic resonance imaging, Morphological parameters

## Abstract

**Background:**

To investigate the morphological parameters of the vastus medialis obliquus (VMO) muscle and delineate its importance in the maintenance of patellofemoral joint stability.

**Methods:**

The magnetic resonance imaging data of seventy-five knees (fifty-four patients) with recurrent lateral patella dislocation (LPD) and seventy-five knees (seventy patients) without recurrent LPD were retrospectively analysed. Five morphological parameters related to the VMO (elevation in the sagittal plane and coronal plane, craniocaudal extent, muscle-fibre angulation, cross-sectional area ratio) and two patella tilt parameters (patella tilt angle, bisect offset ratio) were measured in MR images. The independent-samples t test or chi-square test was used for statistical comparisons.

**Results:**

The mean ages of the patients in the recurrent LPD group and control group were 22.1 ± 9.9 years and 24.0 ± 6.5 years, respectively. Eighteen out of seventy-five (24%) patients MRI showed VMO injuries. Compared with the control group, the patients with recurrent LPD showed significantly higher sagittal VMO elevation (10.4 ± 2.3 mm vs. 4.1 ± 1.9 mm), coronal VMO elevation (15.9 ± 5.7 mm vs. 3.9 ± 3.7 mm), muscle-fibre angulation (35.4 ± 8.0° vs. 27.9 ± 6.3°), patella tilt angle (25.9 ± 10.7° vs. 9.1 ± 5.2°), and bisect offset ratio values (0.9 ± 0.3 vs. 0.5 ± 0.1) and significantly lower craniocaudal extent (13.7 ± 5.3 mm vs. 16.7 ± 5.1 mm) and cross-sectional area ratio values (0.05 ± 0.02 vs. 0.07 ± 0.02).

**Conclusions:**

The results showed that abnormalities in the VMO and patella tilt were clearly present in recurrent LPD patients compared with normal people.

## Background

Recurrent lateral patellar dislocation (LPD) is usually secondary to primary acute patellar dislocation and mostly occurs in young people aged 10–17 years [1]. The incidence of primary acute patella dislocation in the general population is 7–49 cases per 100,000 [2, 3]. With nonoperative management, the rate of recurrent LPD after acute patellar dislocation has been reported to be as high as 44% [4]. Recurrent LPD often causes symptoms including persistent pain, knee weakness and mechanical limitations [5].

Patellofemoral joint stability is maintained by both bone and soft tissue stabilizers. Numerous studies have investigated the effect of osseous factors on LPD, but the influence of soft tissue factors is still being explored. Soft tissues can be considered either active structure stabilizers (quadriceps femoris) or passive structure stabilizers (ligaments), which stabilize the patellofemoral joint together during knee flexion [6]. The medial patellofemoral ligament (MPFL) accounts for 50–60% of the total limiting force against LPD, which is generally considered to be the most important soft tissue in the medial region of the patellofemoral joint [1, 7, 8]. However, several scholars have confirmed that the quadriceps muscle also plays an imperative role in maintaining the stability of the patella [9–12]. The vastus medialis obliquus (VMO) muscle seems to be an important dynamic stabilizer for neutralizing the lateral force of the patella, and its importance is gradually being recognized [9, 13, 14]. In general, the lateral pull of the larger vastus lateralis (VL) is counterbalanced by the force of the VMO to ensure patellar stability. When there is an imbalance, abnormal lateral tracking of the patella may occur. The disruption of this mechanical balance between the VMO and VL has frequently been attributed to an insufficiency of the VMO secondary to atrophy and hypoplasia [15]. Due to the anatomical relationship and characteristics of the MPFL and VMO, MPFL tears are usually accompanied by VMO injuries, while most MPFL tears occur on the femur side. This may cause the femoral attachment point of the VMO to elevate in the sagittal and coronal planes, decreasing the dynamic medial stabilizing force. Unfortunately, VMO injuries occurs in approximately 45–93% of primary patella dislocation patients [16], which may lead to secondary atrophy of the VMO.

To our knowledge, no studies have comprehensively described the morphological characteristics of the VMO in patients with recurrent LPD. Magnetic resonance imaging (MRI) is the gold standard for assessing soft tissue and can clearly show the contours of muscles [17]. Therefore, the purpose of our study was to investigate the difference in VMO-related morphological parameters assessed by MRI between patients with recurrent LPD and a control group.

## Materials and methods

All patients undergoing MRI examinations in our hospital from June 2018 to June 2020 were selected. First, the two keywords of “patella dislocation” and “no obvious abnormality of knee joint” were used to search MR reports in the picture archiving and communications system (PACS) workstation (Centricity, GE Healthcare, St. Gilles, United Kingdom) to initially screen patients. Then, on the basis of both their medical histories and previous medical records. Two head doctors with more than three years of work experience in joint and sports medicine screened the subjects according to the following inclusion and exclusion criteria. A total of fifty-four patients with recurrent LPD (seventy-five knees) and seventy controls (seventy-five knees) were enrolled. Moreover, age and sex were matched as closely as possible between the two groups.

The inclusion and exclusion criteria for the patients with recurrent LPD were as follows:

Inclusion criteria: (1) Recurrent LPD was diagnosed by two senior doctors in the joint and sports medicine department according to the patient's history, physical examination findings and MRI findings. (2) The patient was not previously treated in the rehabilitation department or receive any special training related to strengthening the quadriceps muscles. (3) MRI images were taken within 10 days after the recurrence of LPD.

Exclusion criteria: (1) Patients with primary patellar dislocation. (2) Traumatic patellar dislocation occurred as a result of direct trauma to the medial patella or a fall onto the knee joint with concomitant patellar dislocation. (3) Patients with any preexisting knee disorders, previously underwent knee surgery, had a fracture of the distal femur or tibial head, or had a multi-ligament injury. ④ Patients with history of a neuromuscular disease (e.g., polio). ⑤ Patients with obvious effusion of the knee joint.

The inclusion and exclusion criteria for the control group were as follows: MRI examination of the knee was performed for people to exclude diseases because of knee discomfort and no significant structural damage (e.g., fractures) or anatomical abnormalities (e.g., osteoarthritis) was reported.

Sagittal, coronal, and transverse MR images were obtained in all patients. Two doctors in joint and sports medicine measured the following five parameters related to the VMO (elevation on sagittal plane and coronal plane, craniocaudal extent, muscle-fibre angulation, cross-sectional area ratio) and two patella tilt parameters (patella tilt angle, bisect offset ratio) in both groups. The type of femoral trochlear dysplasia present in each patient was recorded according to the classification system reported by Dejour et al. [18] and Lippacher et al. [19] on axial MR images: type A, shallow trochlea and a subchondral sulcus angle > 145 degrees; type B, flat or convex trochlea; type C, asymmetry of trochlear facets with a hypoplastic medial facet; type D, asymmetry of trochlear facets or cliff pattern, it was further categorized as normal or low-grade (type A), or high-grade dysplasia (type B, C, or D). Moreover, the diagnosis of the VMO injury was recorded according to the criteria reported by Elias et al. [20]. Except for the cross-sectional area, which was calculated by ImageJ freeware, the parameters were measured by the PACS workstation. All the parameters were repeatedly measured within an interval of two weeks. The MRI (Philips MR Systems Ingenia 3.0T, Andover, Massachusetts) protocols used in our hospital were described in our previous study [21]. All patients were in a supine position, with a standard knee coil center level against the lower edge of the patella. The knee and hip joint naturally extended, and the feet were braced to prevent any movement. Our MRI protocol includes: (1) coronal proton density weighted spectral attenuated inversion recovery (PDW-SPAIR) MR images [repetition time msec (TR)/echo time msec (TE) 1940/30, field of view (FOV) 220 mm × 179 mm, matrix 368 × 245, slice thickness 3 mm, sections per slab 21]; (2) transverse PDW-SPAIR MR images (TR/TE 2036/30, FOV 169 mm × 189 mm, matrix 344 × 264, slice thickness 4 mm, act slice gap 0.4 mm, sections per slab 24); (3) sagittal T1-weighted aTSE (turbo spin-echo) MR images (TR/TE 694/12, FOV 160 mm × 160 mm, matrix 308 × 240, slice thickness 3 mm, act slice gap 0.3 mm, sections per slab 24); (4) sagittal proton density weighted spectral inversion recovery (PDW-SPIR) MR images (TR/TE 1,554/30, FOV 160 × 160 mm, matrix 292 × 231, slice thickness 3 mm, act slice gap 0.3 mm, sections per slab 24).

### MR measurements

#### The measurement of VMO elevation

**Fig.1:**
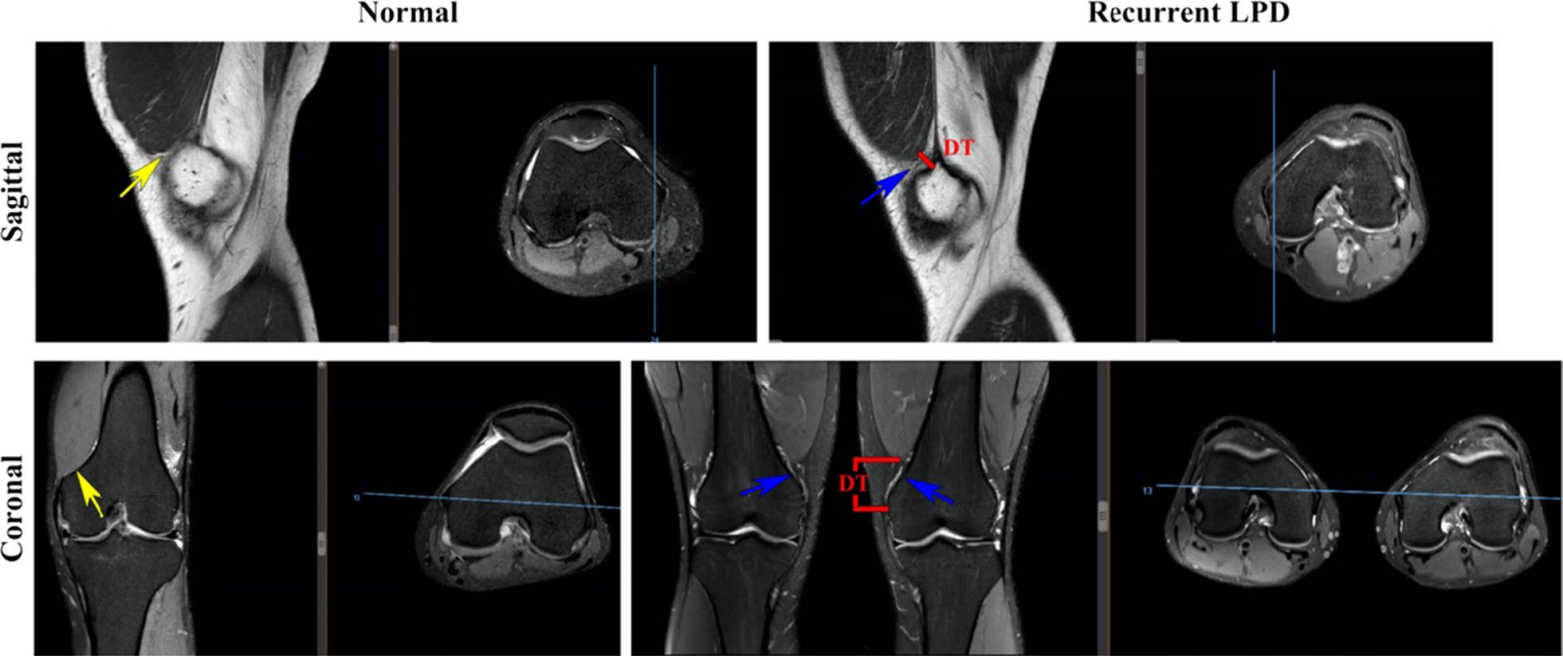
The measurement of VMO elevation in the control and LPD groups. DT represents the elevation distance of the vastus medialis obliquus (VMO) in the sagittal and coronal planes. Under normal conditions, the VMO was attached to the medial femoral condyle in the sagittal and coronal images (indicated by the yellow arrow), but it was significantly elevated in the recurrent lateral patellar dislocation (LPD) patients (indicated by the blue arrow)

The VMO elevation was measured in the sagittal and coronal planes according to Zhang et al.'s [22] measurement method. In brief, the transverse slice in which the adductor tubercle could clearly be seen was defined as the optimally measurable slice, as indicated by a blue line. In this transverse image, the corresponding sagittal and coronal planes were identified (Fig. 1).

On the selected sagittal slice, the apex of the anterosuperior border of the bone cortex of the adductor tubercle was set as the starting point. VMO elevation was defined as the shortest distance from the starting point extending obliquely to the inferior edge of the muscle belly. On the selected coronal slice, the apex of the medial superior border of the adductor tubercle was set as the starting point. VMO elevation in the coronal plane was defined as the vertical distance from the starting point to the inferior margin of the VMO muscle (Fig. 1).

#### The measurement of muscle-fibre angulation and craniocaudal extent of the VMO

**Fig. 2:**
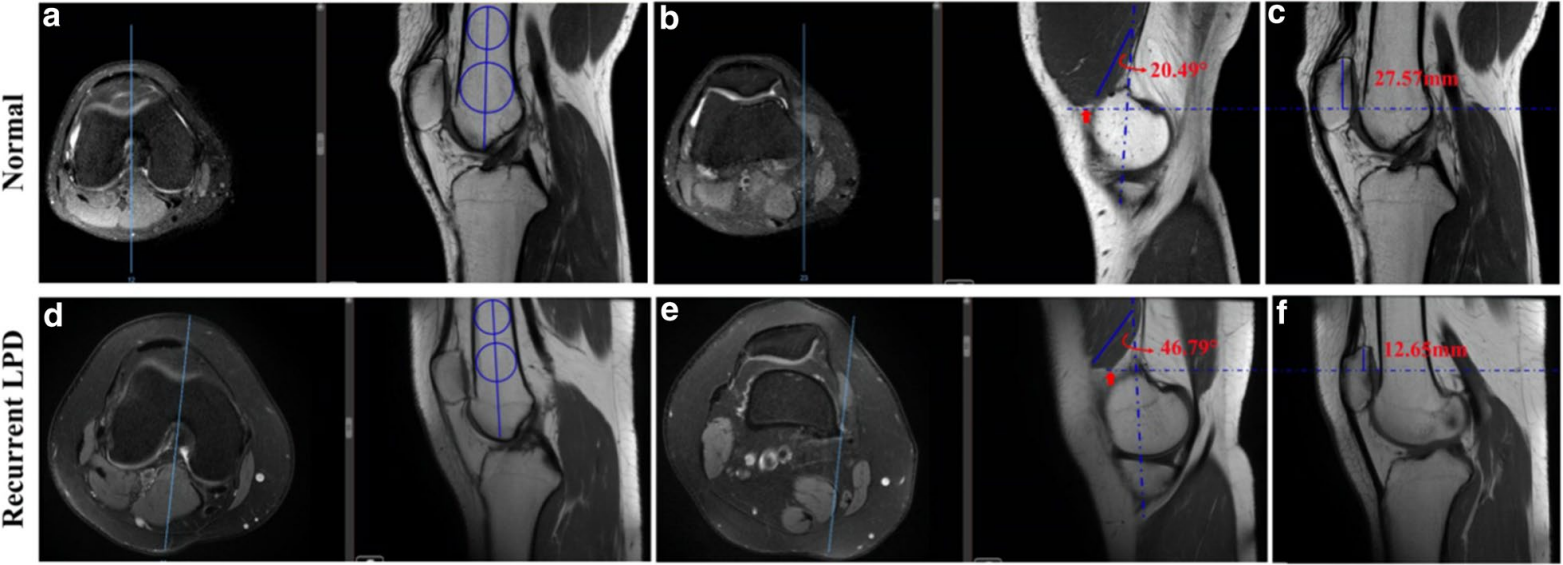
The measurement of muscle fibre angulation and craniocaudal extent of the VMO in the control and recurrent LPD groups. **a**, **d** Images showing the plane in which the “Roman arch” was most obvious; the longitudinal axis of the femoral shaft was identified; **b**, **e** The adductor tubercle was located in the medial margin of the femur in the transverse slice; the red dots indicate the lowest point of the VMO, and the VMO muscle-fibre angulation in the controls and LPD patients were 20.49° and 46.79°; **e**, **f** The magnitudes of craniocaudal extent of the VMO in the controls and LPD patients were 27.57 mm and 12.65 mm, respectively

First, the “Roman arch” was most obvious in the axial plane, and the corresponding sagittal slice was selected (Fig. 2a, d). Two concentric circles were drawn on the proximal and distal sides of the femur, and the line passing through two centers was taken as the longitudinal axis of the femoral shaft. Second, the adductor tubercle was found on the transverse slice, and the corresponding sagittal slice was located to determine the lowest point of the VMO. The VMO muscle-fibre angulation, the angle between the VMO muscle-fibre and the longitudinal axis of femoral shaft, was measured in the sagittal plane (Fig. 2b, e). The lowest point of VMO was located in this plane, and the corresponding horizontal line was established in the sagittal plane central to the patella longitudinal axis. The craniocaudal extent of the VMO was defined as the vertical distance from this horizontal line to the proximal patellar pole (Fig. 2c, f).

#### The measurement of the cross-sectional area ratio of the VMO

**Fig. 3:**
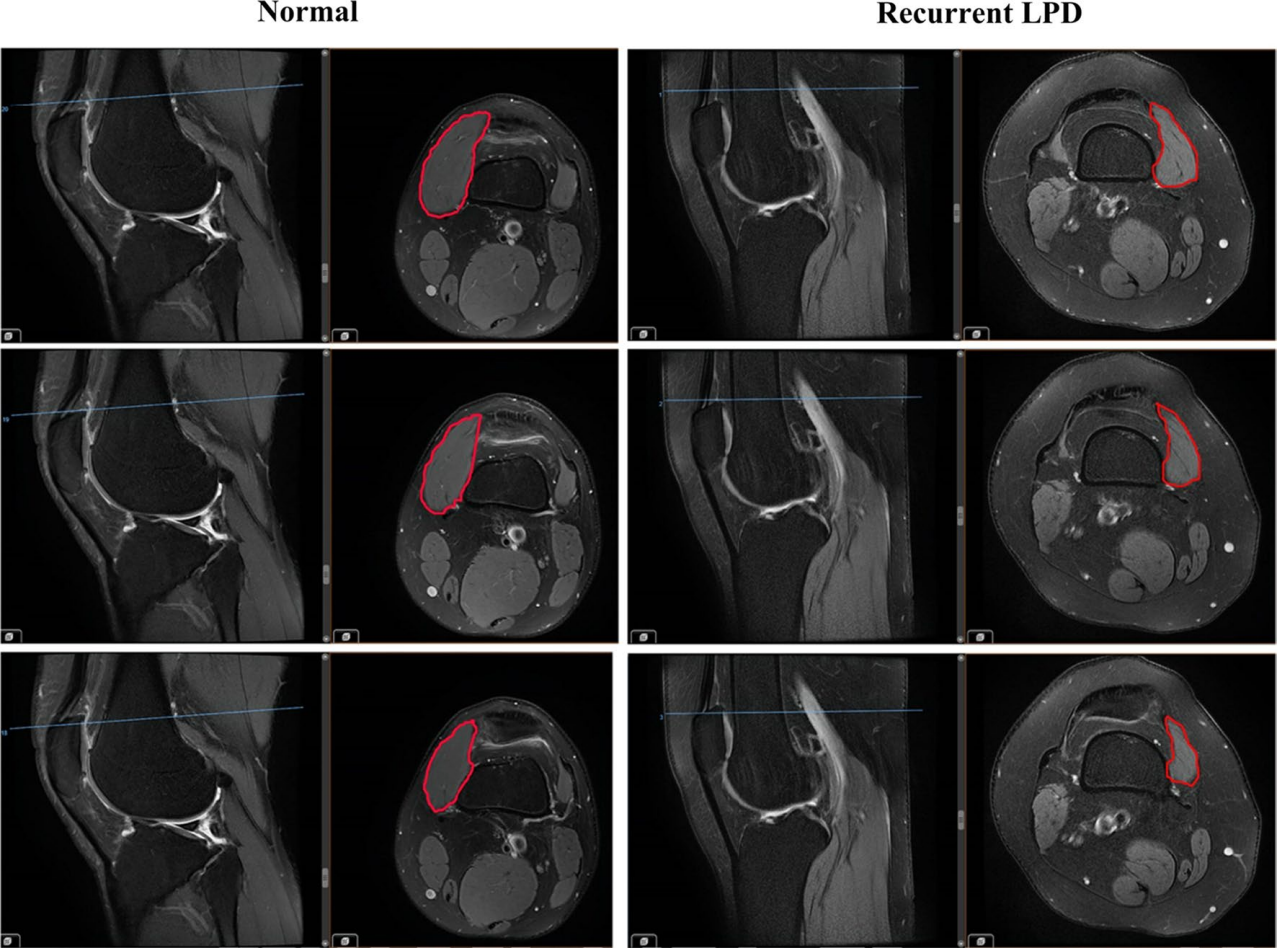
The measurement of the cross-sectional area ratio of the VMO in the control and recurrent LPD groups. The three solid blue lines correspond to the three adjacent transverse slices, and the part indicated by the red line is the cross-sectional area of the VMO

According to the method introduced by Balcarek et al. [23]. First, the longitudinal axis of the patella was established in the central sagittal plane. In this sagittal image, the corresponding transverse slice located at the proximal patellar pole and the adjacent slice located above and below this reference slice were identified. Then the cross-sectional area of the VMO and the whole thigh were calculated on these three slices respectively (Fig. 3). The cross-sectional area ratio of the VMO was defined as the ratio between the cross-sectional area of the VMO and the whole thigh. Finally, the mean cross-sectional area ratio among the three slices was obtained.

#### The measurement of the patella tilt angle and patella offset index

**Fig. 4:**
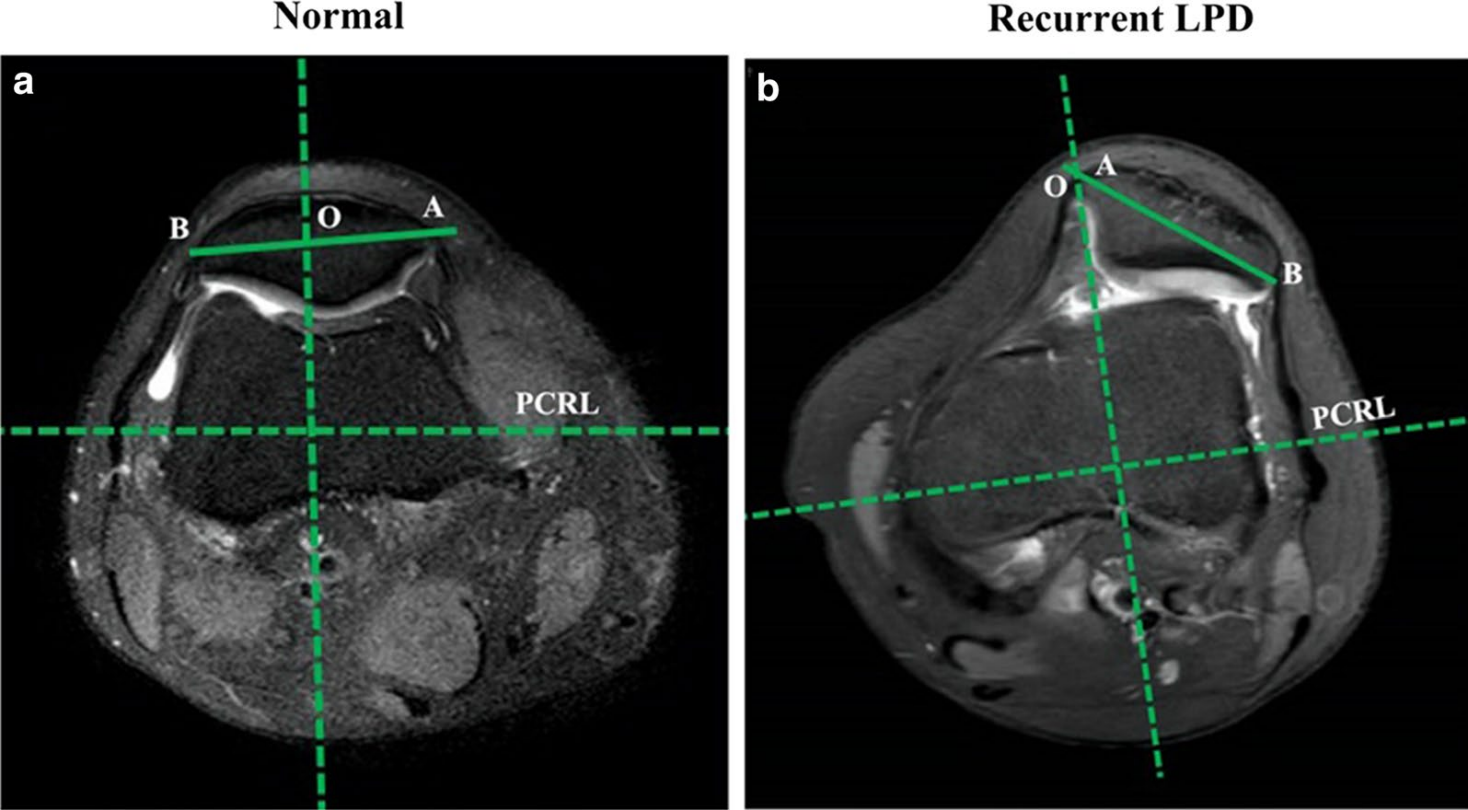
The measurement of the patella tilt angle and bisect offset index in the control and recurrent LPD groups. The posterior condylar reference line (PCRL) was drawn tangent to the posterior femoral condyles. The tilt angle was measured as the angle between the PCRL (dashed line) and the line along the width of the maximal patella (solid line). A line was drawn through the deepest portion of the trochlear groove and perpendicular to the PCRL. The intersection of this line and the maximal patella width line was defined as point O. In the transverse plane of the widest layer of the patella, the innermost point of the patella was defined as point A, and the outermost point was defined as point B. The ratio of OB/AB was defined as the bisect offset ratio

The transverse plane, which allows the visualization of the intact “Roman arch” and posterior femoral condyles, was selected. The posterior condylar reference line was drawn tangent to the posterior femoral condyles. The patella tilt angle was formed by the line along the width of the maximal patella and the line along the posterior femoral condyle (Fig. 4).

According to the method described by Christopher et al. [24] and Callaghan et al. [25], a line was drawn through the deepest portion of the trochlear groove and perpendicular to the posterior condylar reference line. The intersection of this line and the line along the width of the maximal patella was defined as point O. In the transverse plane of the widest layer of the patella, the innermost point of the patella was defined as point A, and the outermost point was defined as point B (Fig. 4). The ratio of OB/AB was defined as the bisect offset ratio.

### Statistical analysis

SPSS 22.0 (IBM Corp. Released 2013. IBM SPSS Statistics for Windows. Armonk, NY: IBM Corp) was used to assess the relevant data. All parameters are presented as the mean ± standard deviation. The continuous and categorical variables were compared between the two groups were analyzed by the independent-samples t test and chi-square test, respectively. *P* < 0.05 was considered statistically significant. Moreover, the intraclass correlation coefficient (ICC) was also analysed for duplicated measurements taken by two observers.

## Results

Fifty-four patients (seventy-five knees) diagnosed with recurrent LPD were enrolled in our study. The study group consisted of eighteen males and thirty-six females. The average age of the patients was 22.11 ± 9.87 years (range, 12–45 years), and their average BMI was 24.1 ± 3.6 kg/m^2^. In addition, seventy controls (seventy-five knees) were recruited. The baseline characteristics of the two groups are presented in Table 1. Compared with the control group, the recurrent LPD group showed more severe femoral trochlear dysplasia, as indicated by a higher grade (Table 1). Eighteen out of seventy-five (24%) patients MRI showed VMO injuries, and VMO tear was present in only one patients, oedema signal was observed in thirteen patients and haemorrhage signal was found in the other four patients. Additionally, the intraclass correlation coefficients indicated excellent inter- and intraobserver agreement for all the variables (> 0.75).

**Table 1:** The basic characteristics of the patients in the two groups

Group	Age, mean ± SD (range), y	Sex (male/female)	BMI, mean ± SD (kg/m^2^)	Trochlear dysplasia (n)		
				Normal	Low-grade	High-grade
Control	24.0 ± 6.5 (19–38)	25/45	23.3 ± 2.6	57	18	0
Recurrent LPD	23.1 ± 9.9 (12–45)	18/36	24.1 ± 3.6	0	16	59
*p*	> .05	> .05	> .05	< .05	> .05	< .05

*LPD* lateral patellar dislocation

As shown in Fig. 1, the VMO was attached to the medial femoral condyle in the sagittal and coronal sectional images in the control group, but it was significantly elevated in the LPD patients. In addition, the patellofemoral joint was in good alignment in the controls. The mean magnitudes of sagittal and mean coronal VMO elevations were significantly higher in the recurrent LPD group than in the control group (10.4 ± 2.3 mm vs. 4.1 ± 1.9 mm, 15.9 ± 5.7 mm vs. 3.9 ± 3.7 mm, respectively). Compared with the control group, the recurrent LPD group showed significantly higher muscle-fibre angulation in the VMO (35.4 ± 8.0° vs. 27.9 ± 6.3°). However, the craniocaudal extent of the VMO was significantly smaller in the LPD group than in the control group (13.7 ± 5.3 mm vs. 16.7 ± 5.1 mm). The average cross‑sectional area ratio of the VMO was 5% in the recurrent LPD group and 7% in the control group (Table 2).

**Table 2:** Comparison of the study group and recurrent LPD group

	Mean VMO elevation (mm)		Muscle-fibre angulation (°)	Craniocaudal extent (mm)	Cross-sectional area ratio	Patella tilt angle (°)	Bisect offset ratio
	Sagittal	Coronal					
Control	4.1 ± 1.9	3.9 ± 3.7	27.9 ± 6.3	16.7 ± 5.1	0.07 ± 0.02	9.1 ± 5.2	0.54 ± 0.06
Recurrent LPD	10.4 ± 2.3	15.9 ± 5.7	35.4 ± 8.0	13.7 ± 5.3	0.05 ± 0.02	25.9 ± 10.7	0.97 ± 0.33
*P* value	0.00	0.00	0.00	0.005	0.000	0.00	0.00

*LPD* Lateral patellar dislocation, *VMO* vastus medialis obliquus

As shown in Fig. 4, normally, the line that passes through the deepest portion of the trochlear groove and was perpendicular to the posterior condylar reference line passed through near the midpoint of the widest plane of the patella (Fig. 4a). However, obvious patellar tilt and displacement were observed in the recurrent LPD group (Fig. 4b). Compared with the control group, the recurrent LPD group showed a significantly larger patella tilt angle (25.9 ± 10.7° vs. 9.1 ± 5.2°); similarly, the bisect offset ratio of the LPD group was significantly higher than that of the control group (0.97 ± 0.33 vs. 0.54 ± 0.06).

## Discussion

It is generally believed that patella maltracking usually manifests as the subluxation and outward displacement of the patella, which is mainly measured by the patella tilt angle and bisect offset ratio. The patella tilt angle is used to describe the degree of inclination of the patella, which is regarded as the most sensitive indicator to identify patella instability [26]. Lateral displacement of the patella was described by the bisect offset ratio, which was defined as the percentage of lateral width of the total patellar width [27]. Therefore, the patella tilt angle and bisect offset ratio were used to assess the patella position in patients with recurrent LPD in our study. As in the studies with conducted by Charles et al. [28] and Escala et al. [26], our study revealed a statistically significant difference in the patellar tilt angle between the control persons (9°) and recurrent LPD patients (25°). Moreover, another indicator of lateral tilt, the bisect offset ratio, showed a significant difference in two groups: the values were 0.54 and 0.97 in the control and LPD groups, respectively. The results mentioned above confirmed the existence of patellar inclination in recurrent LPD patients in our investigation. Femoral trochlear dysplasia has been recognized as a risk factor for patellar dislocation. The latest literature indicated that patellofemoral kinematics, stability, contact pressure, and contact area are significantly affected by trochlear dysplasia [29]. Trochleoplasty has also been shown to be effective in patients with severe trochlear dysplasia [30]. Our results also showed that high-grade trochlear dysplasia was more common in patients with recurrent LPD.

The VMO acts as a vital dynamic stabilizing device to limit the tendency of patellar dislocation. Anatomically, the VMO is basically perpendicular to the longitudinal axis of the patella sagittal position, thereby enhancing the stability of the patella [31]. The patella does not contact the trochlea at the beginning of knee flexion, and the tendency for patella dislocation is limited by the VMO and MPFL. An in vitro study revealed that when the VMO is weak, patellar displacement increases throughout 0–15° of knee flexion [32]. Throughout 20°–90° of knee flexion, VMO relaxation can reduce the resistance of the patella lateral displacement by 30% [33]. Therefore, we consider that patellar tilt or lateral dislocation may be induced by abnormalities in the VMO. Until now, there has been no consensus on the role of the VMO in the stabilization of the patellofemoral joint, and some scholars have doubted that patellar instability is related to the VMO [34, 35]. Nevertheless, more studies have shown that the VMO significantly affects patellofemoral instability. Pattyn et al. [15] suggested that VMO atrophy is present in patients with patellofemoral pain and is a contributing factor to patellofemoral pain syndrome. Moreover, the idea that the functional status of the VMO is closely related to recurrent LPD was verified in a diffusion tensor imaging study [36]. In a 2-year follow-up study, MPFL reconstructive surgery without VMO repair had no significant effect on re-dislocation in patients with primary patella dislocation compared with conservative treatment [37], which suggested that MPFL reconstruction combined with VMO repair may yield better postoperative outcomes. Zhang et al. [22] also suggested that more attention should be paid to the VMO, especially in those patients with complete femoral-side injuries. Hence, in preoperative evaluations, surgeons should carefully assess the injury conditions of the MPFL and VMO, and individualized treatment should be adopted for each patient.

In this study, we used elevation in the sagittal plane and coronal plane, cross-sectional area, muscle-fibre angulation and craniocaudal extent to comprehensively evaluate the morphological changes of the VMO in recurrent LPD patients. The VMO is originally attached intimately to the patella together with MPFL, and the presence of pathological elevation means that the VMO is no longer connected at the original attachment point to the patella or femur. The loss of the firm attachment to the distal origin, resulting in large sagittal and coronal elevations of the torn VMO muscle, may decrease the dynamic medial stabilizing force. This can be manifested by the elevation of the VMO in the coronal and sagittal planes, as observed in MRI scans, which may lead to a reduction in the limiting force of the VMO on the medial patellofemoral joint, thus increasing the extent of lateral inclination of the patella. This conclusion was confirmed in a cadaver study by Goh et al. [38], and comparisons showed that the VMO tension loss causes an increase in lateral displacement of the patella and increases the stress of the lateral patellar facet during knee flexion. Our results suggest that compared with individuals in the control group, patients with recurrent LPD have an VMO that is elevated by an average of about approximately 6 mm and 12 mm in the sagittal and coronal planes, respectively. These results indicated that the VMO is significantly elevated in recurrent LPD patients. However, the VMO was significantly elevated more in Zhang et al.’s study [22] than in our study. Elias et al. [20] found that approximately 55% of patients with acute LPD had significant effusion; thus, the average elevation of the VMO in the coronal plane was 2.2 cm, which was also larger than that observed in our results, while the elevation in the sagittal plane was not noted. We speculate that this inconsistency may have been caused by massive effusions accumulating in the cavity after the first acute LPD, which leads an obvious degree of elevation of the VMO.

In addition, muscle-fibre angulation and craniocaudal extent may also have an influence on the tension of the VMO, and a cadaver study has shown that the absence of VMO tension leads to a lateral shift of the patella [38]. Our results showed that the muscle-fibre angulation and craniocaudal extent of the VMO significantly differed between the two groups, the muscle-fibre angle was larger by an average of 12° and craniocaudal extent was smaller by 3 mm in the LPD group than in the control group. In our investigation, the average craniocaudal extent was 13.7 mm in patients with recurrent LPD, which was consistent with the average of 14 mm reported in Balcarek et al.’s study [23]. Previous cadaver studies have indicated that muscle-fibre angulation of the VMO in normal people ranges from 42° to 52° [31]; however, the average muscle-fibre angulation in the control group and recurrent LPD patients were 27° and 35° in our study, respectively. Balcarek et al. [23] found that the muscle-fibre angulation in the control group was 44°–48°, the prevalence of recurrent LPD females subjects in our study was 33.3% (18/54) while in their study was 50% and there was also a difference in the BMI of the population, so we think this discrepancy might be related to the study population. Moreover, in the LPD group in their study, this angle was smaller than that in the control group (no significant statistical difference), but we obtained the opposite result. In fact, it seems plausible that the VMO will tear or injure in femoral attachment points after LPD, causing the muscle to move away from adductor tubercle. Therefore, the muscle fibres of the VMO will shift forward and upward relative to the longitudinal axis of femur in the sagittal plane, which will inevitably lead to an increase rather than a decrease in muscle-fibre angulation (Fig. 2b, e).To date, no similar studies have been conducted, so the normal range of muscle-fibre angulation of the VMO remains to be studied further.

It has been proven that the cross-sectional area can be used to assess the force-producing ability of muscle and can be measured on MR images [39, 40]. To simulate the three-dimensional structure of the muscle, three adjacent layers of the upper edge of the patella were selected to calculate the cross-sectional area of the VMO (Fig. 3). Furthermore, we calculated the ratio between the cross-sectional area of the VMO and the whole thigh to obtain the volume proportion of the VMO and minimize individual differences. As a result, the ratio of the VMO cross-sectional area of the corresponding thigh area in the recurrent LPD group was significantly lower than that in the control group. Both Balcarek et al. [23] and Liu et al. [36] found that the cross‑sectional area of the VMO showed a decreasing trend in patients with recurrent PLD, but the differences between these patients and healthy volunteers were not significant; this lack of statistical significance may be related to the small sample size, as there were only 30 cases of LPD group in both studies. Another reason may be that we used the ratio to personalize the cross-sectional areas, which are more likely to show significant differences.

At present, there are no widely accepted quantitative indicators to evaluate VMO injuries on MRI. Zhang et al. [22] found that probability of injury of the VMO was 47.7% in patients with first patellar dislocation, indicating that nearly half of the patients with acute first LPD had a VMO injury. In this study, the recurrent LPD patients we included did not have a history of acute trauma in the short term, and the proportion of VMO injuries in the recurrent LPD group was only 24% (18/75). Therefore, in patients with recurrent LPD, it is difficult to determine whether the VMO is injured on the basis of only the presence of haemorrhage and oedema in MRI images. We attempted to use the above five quantitative indicators to evaluate VMO abnormalities, which can be caused by congenital factors or incomplete healing due to the failure to seek treatment after the initial dislocation.

Nonetheless, the results above should be interpreted with consideration of the limitations of this investigation. First, as this study was retrospective, the patients with recurrent LPD had obvious anatomical abnormalities, so the assessors could not be blinded to the diagnoses of the patients during the assessments. Second, our study indicated only that the VMO is abnormal in recurrent LPD patients, while it did not reveal whether the abnormality is a contributing factor or secondary to recurrent LPD. Third, the static morphological characteristics of only the VMO were studied, and we did not assess the vastus lateralis muscle; the balance between the two muscles may be important for evaluating the role of the VMO in maintaining patella stability. Moreover, recurrent LPD is often the result of multiple factors. To determine the role of the VMO in recurrent LPD patients, other factors (e.g., trochlear dysplasia, patella alta, and an increased tibial tuberosity-trochlear groove distance) for patellar dislocation were not analysed, so a more detailed and comprehensive assessment remains to be completed.

## Conclusions

In conclusion, the results showed that abnormalities of the VMO were clearly present in recurrent LPD patients compared with normal people. MRI was used to comprehensively evaluate the morphological parameters of the VMO in patients with recurrent LPD, and the presence of VMO elevation in the coronal and sagittal planes, a low cross-sectional area ratio, a high muscle-fibre angulation and a small craniocaudal extent may be related to patellar inclination. Based on the results of previous biomechanical and clinical studies, we summarized the methods for evaluating the VMO by MRI, which is helpful for clinicians to identify VMO atrophy by objective morphological parameters. Moreover, by using the individualized index (cross-sectional area ratio of the VMO and bisect offset ratio), we can better to reduce the measuring errors and evaluated the abnormalities of the VMO and patella deviation.

## Data Availability

The data for this study are provided in the Tables of the main paper. Due to patient privacy protection, other additional materials and data are not publicly available but are available from the corresponding author on reasonable request.

## Abbreviations

FOV: Field of view
ICC: Intraclass correlation coefficient
LPD: Lateral patella dislocation
MRI: Magnetic resonance imaging
MPFL: Medial patellofemoral ligament
PACS: Picture archiving and communications system
PDW-SPAIR: Proton density weighted spectral attenuated inversion recovery
PDW-SPIR: Pproton density weighted spectral inversion recovery
TR: Time msec
TE: Time msec
TSE: Turbo spin-echo
VMO: Vastus medialis obliquus
VL: Vastus lateralis
